# Inter-organ communication: pathways and targets to cardioprotection and neuro-protection. A report from the 12th Hatter Cardiovascular Institute workshop

**DOI:** 10.1007/s00395-024-01094-6

**Published:** 2024-12-16

**Authors:** L. Pearce, C. Galán-Arriola, R. M. Bell, R. D. Carr, J. Cunningham, S. M. Davidson, A. K. Ghosh, S. Giesz, P. Golforoush, A. V. Gourine, D. M. Hermann, G. Heusch, B. Ibanez, S. Beikoghli Kalkhoran, S. Lecour, K. Lukhna, M. Ntsekhe, M. N. Sack, R. J. Unwin, G. Vilahur, J. M. Walker, D. M. Yellon

**Affiliations:** 1 The Hatter Cardiovascular Institute, University College London, 67 Chenies Mews, London, WC1E 6HX UK; 2 Centro Nacional de Investigaciones Cardiovasculares (CNIC), Madrid, Spain; 3 School of Biomedical Sciences, Ulster University, Coleraine, UK; 4 Centre for Nephrology, University College London, London, UK; 5 Centre for Cardiovascular and Metabolic Neuroscience, Neuroscience, Physiology and Pharmacology, University College London, London, UK; 6 Chair of Vascular Neurology, Dementia and Ageing Research, University Hospital Essen, University of Duisburg-Essen, Essen, Germany; 7 Institute for Pathophysiology, West German Heart and Vascular Center, University of Duisburg-Essen, Essen, Germany; 8 CIBER de Enfermedades Cardiovasculares (CIBERCV), Madrid, Spain; 9 IIS-Fundación Jiménez Díaz Hospital, Madrid, Spain; 10 University of Cape Town, Cape Town, South Africa; 11 Laboratory of Mitochondrial Biology and Metabolism, NHLBI, National Institutes of Health, Bethesda, MD USA; 12 Institut de Recerca Sant Pau, IIB-Sant Pau, Hospital de la Santa Creu i Sant Pau, CIBERCV, Barcelona, Spain

**Keywords:** Brain, Cardio-oncology, Cytoprotection, Heart, Ischaemia, Kidney, Reperfusion, Signalling

## Abstract

A long-standing aim in the setting of various pathologies including acute myocardial infarction, chronic kidney disease (CKD), and ischaemic stroke, has been to identify successful approaches to augment cellular and organ protection. Although the continual evolution and refinement of ideas over the past few decades has allowed the field to progress, we are yet to realise successful clinical translation of this concept. The 12th Hatter Cardiovascular Workshop identified a number of important points and key questions for future research relating to cardio- and neuro-protection and interorgan communication. Specific topics that were discussed include the ‘cardio-metabolic-renal’ axis of organ protection, the parasympathetic signalling hypothesis, the role of the coronary microvasculature in myocardial infarction, the RISK pathway of cardioprotection, extracellular vesicles and the way forward, the future for clinical studies of remote ischaemic conditioning, and new experimental models for cardio-oncology investigations.

## Background

A long-standing aim in the setting of various pathologies including acute myocardial infarction [46], chronic kidney disease (CKD), and ischaemic stroke, has been to identify successful approaches to cellular and organ protection. Although the continual evolution and refinement of ideas over 4 decades has allowed the field to progress, we are yet to realise successful clinical translation of this concept. With this in mind, a group of international investigators in the areas of organ protection (heart, brain, and kidney) gathered in South Africa, for the 12th Biennial Hatter Cardiovascular workshop.

Previous workshops have defined the “critical criteria for success” [10] and have defined ten so-called “commandments of cardioprotection” [11]. However, it is increasingly recognised that the focus on cardioprotection may be too limited, and the broader concept of *interorgan* communication should be considered [11, 85]. The aim of this forum was, therefore, to debate new ideas relating to interorgan communication and protection and discuss possible reasons why previous concepts have failed to achieve clinical translation [44]. Furthermore, whilst previous workshops have concentrated primarily on the cardiovascular system, the 12th workshop expanded this focus to examine pathways and targets relevant to cardio- and neuro-protection. Specific topics that were discussed included the ‘cardio-metabolic-renal’ axis of organ protection, the role of the microvasculature in STEMI, novel phosphatidylinositol 3-kinases (PI3K) agonists for cardioprotection, and new experimental models for cardio-oncology investigations.

A key concept considered was that the heart is part of a multi-organ signalling axis, with both damaging and protective pathways originating from the kidneys, gastrointestinal tract, spleen, and involving the parasympathetic nervous system (PNS). A non-pharmacological approach to protection has been the use of remote ischaemic conditioning (RIC). This is a method of inducing cellular protection in which a distant site (usually limb) is subjected to sequential periods of ischaemia and reperfusion resulting in organ protection [5, 49]. However, given the overall neutral outcomes of large clinical trials of RIC, the participants discussed whether the concept of RIC is “*salvageable”* [50], and explored the potential role of this intervention in the setting of: (i) high- risk STEMI patients, (ii) high-risk STEMI patients with delayed presentation to thrombolysis in remote areas and parts of the world where application of timely PCI is not feasible (i.e.: RIC AFRICA) [71], and (iii) the effects of cancer chemotherapy on cardiovascular outcomes [77]. These concepts are discussed below.

## Cardiometabolic kidney disease and the SGLT2/GLP-1 paradigm

One theme of discussion at the workshop was how to define diseases that increasingly present on a background of multiple co-morbidities [59], since accurate disease diagnosis is required for appropriate treatment. Complicating this process is the fact that many diseases, such as heart failure, stroke, and chronic kidney disease (CKD) are complex syndromes, with multiple diverse aetiologies. For example, the categorisation of heart failure has evolved over the years, previously being defined as “restrictive" or "congestive” based on clinical presentation but is now categorised as heart failure with preserved ejection fraction (HFpEF) or heart failure with reduced ejection fraction (HFrEF) based on functional assessment.

**Fig. 1:**
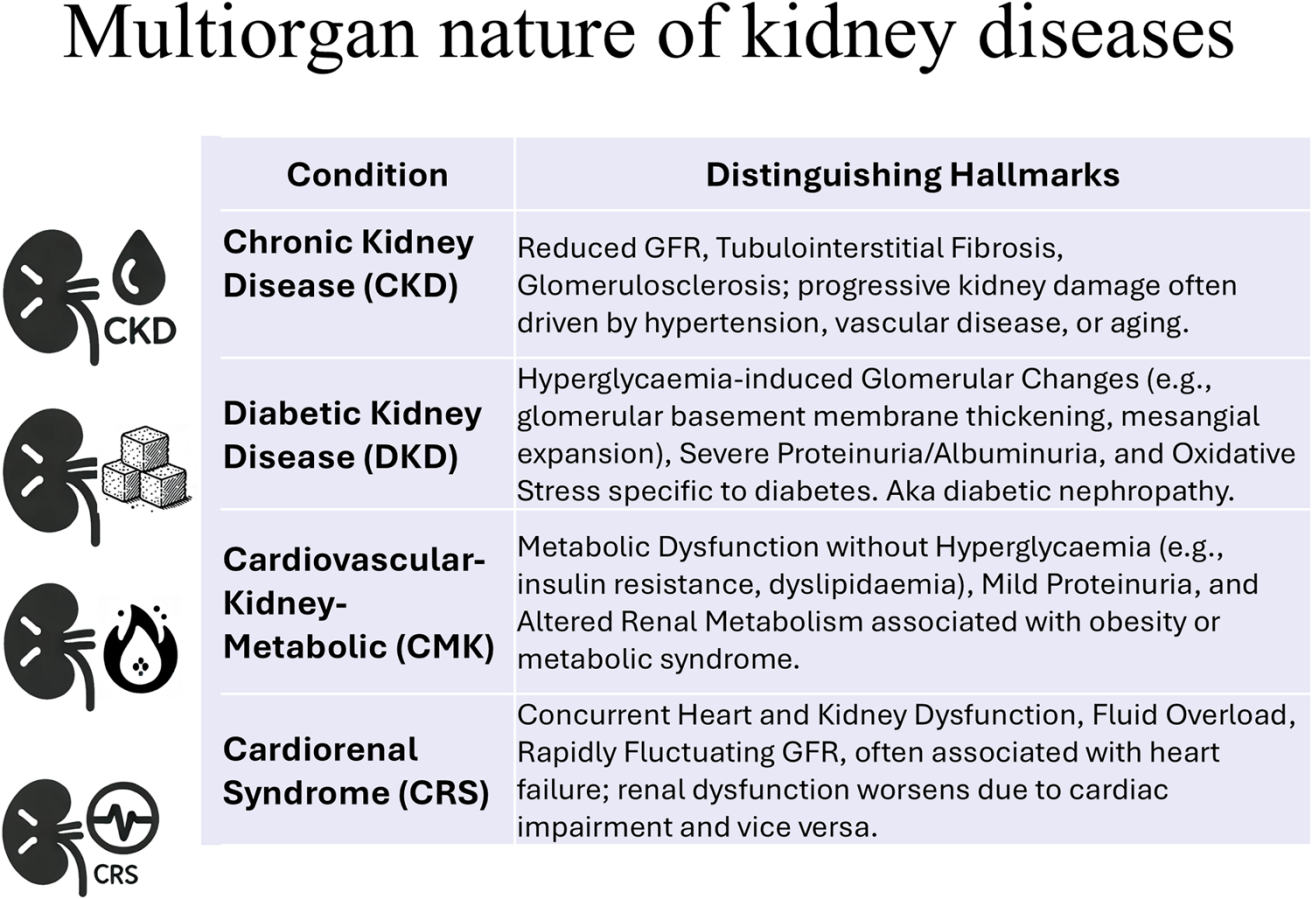
Distinguishing hallmarks of different types of kidney disease that demonstrate their multi-organ nature, and which should be considered in terms of their effects on the heart. GFR, glomerular filtration rate

Chronic kidney disease (CKD) occurs as a gradual, long-term decline in kidney function, often leading to end-stage renal disease (Fig. 1). Cardiorenal syndrome (CRS) is a scenario where heart and kidney dysfunctions exacerbate each other. In diabetic kidney disease (DKD), kidney damage is caused by diabetes, leading to progressive loss of kidney function. Cardiometabolic kidney disease (CMK) is a newly recognised syndrome and a key area for therapeutic intervention, being a significant contributor to overall cardiovascular mortality. It encompasses a variety of clinical syndromes including obesity, diabetes mellitus, CKD, heart failure, coronary artery disease and stroke [79]. CMK has recently been categorised into a four-stage syndrome, progressing from excess adipose tissue (stage 1) all the way to complex multi-organ disease involving hypertension, hypertriglyceridemia, diabetes, heart failure, atrial fibrillation and CKD with albuminuria (stage 4) [79]. In line with the discussions emanating from the workshop, new treatments for CMK target multiple organs, and include sodium glucose co-transporter 2 (SGLT2) inhibitors (such as canagliflozin, dapagliflozin and empagliflozin), and glucagon-like peptide-1 (GLP-1) agonists (such as semaglutide). Both of these classes of drugs are associated with improved cardiorenal outcomes in large-scale clinical trials, despite their original indications as hypoglycaemic agents [3, 40, 81, 84]. Moreover, anti-obesity drugs used in diabetic patients, such as tirzepatide (combined glucose-dependent insulinotropic polypeptide (GIP) and GLP-1RA agonist) lead to improvement in glycaemic control compared to GLP-1 agonist alone [27], and recently demonstrated an improvement in cardiorenal outcomes in a large cohort study [27]. The workshop interpreted these important advances over the past 5 years, as being indicative of a change in basic assumptions from single-organ protection to multi-organ therapies.

The workshop discussed an integrated management model for CMK disease involving the above pharmacological interventions, lifestyle changes, lymphatic therapeutics [24] and improved screening of the above population, with a role for possible biomarkers such as triglyceride-glucose-body mass index (TyG-BMI) [67]. The lymphatic system in CKD and chronic inflammation was felt to be a new area of research that may be integral to injury resolution, and in preventing progression to renal fibrosis. Interestingly, the mechanisms underlying the cardiovascular benefits of the SGLT2 inhibitors remain incompletely understood, considering that SGLT2 is expressed in the kidney and some other organs, but not the heart [52, 92]. This implies there is a role for communication from the kidney to other organs in SGLT2-related cardiovascular protection in the setting of heart failure. This could be via renal afferent signalling and recruitment of vagal parasympathetic protective pathways, as recently suggested [6] (and discussed further below).

The consensus view was that there is a need to develop improved pre-clinical multi-morbid models of kidney injury.

## Novel models of cardiorenal syndrome

A potentially valuable multi-organ disease model that was discussed, as a means of examining how best to protect the myocardium from CKD, is the adenine-induced rat model of CKD with kidney-induced cardiac injury [9]. The potential advantage of this model is that it is an adaptation of the existing adenine-induced model of CKD incorporating an additional period in which adenine is removed from the diet. This allows recovery from any potential acute injury, with the progression of CKD and its consequential effect on the heart. Importantly, animals fed with adenine-supplemented diet have significantly larger infarct sizes following ischaemia/reperfusion, potentially mediated by increased inflammation (CD45+, myeloperoxidase) and capillary rarefaction [9]. An unknown question is whether a single undefined pathological process leads to multi-organ injury in the clinical setting, *or whether these multi-organ phenomena are simply a consequence of ageing and other co-morbidities such as diabetes.*

## The parasympathetic nervous system: a proposal for a common efferent pathway of cardioprotection?

**Fig. 2:**
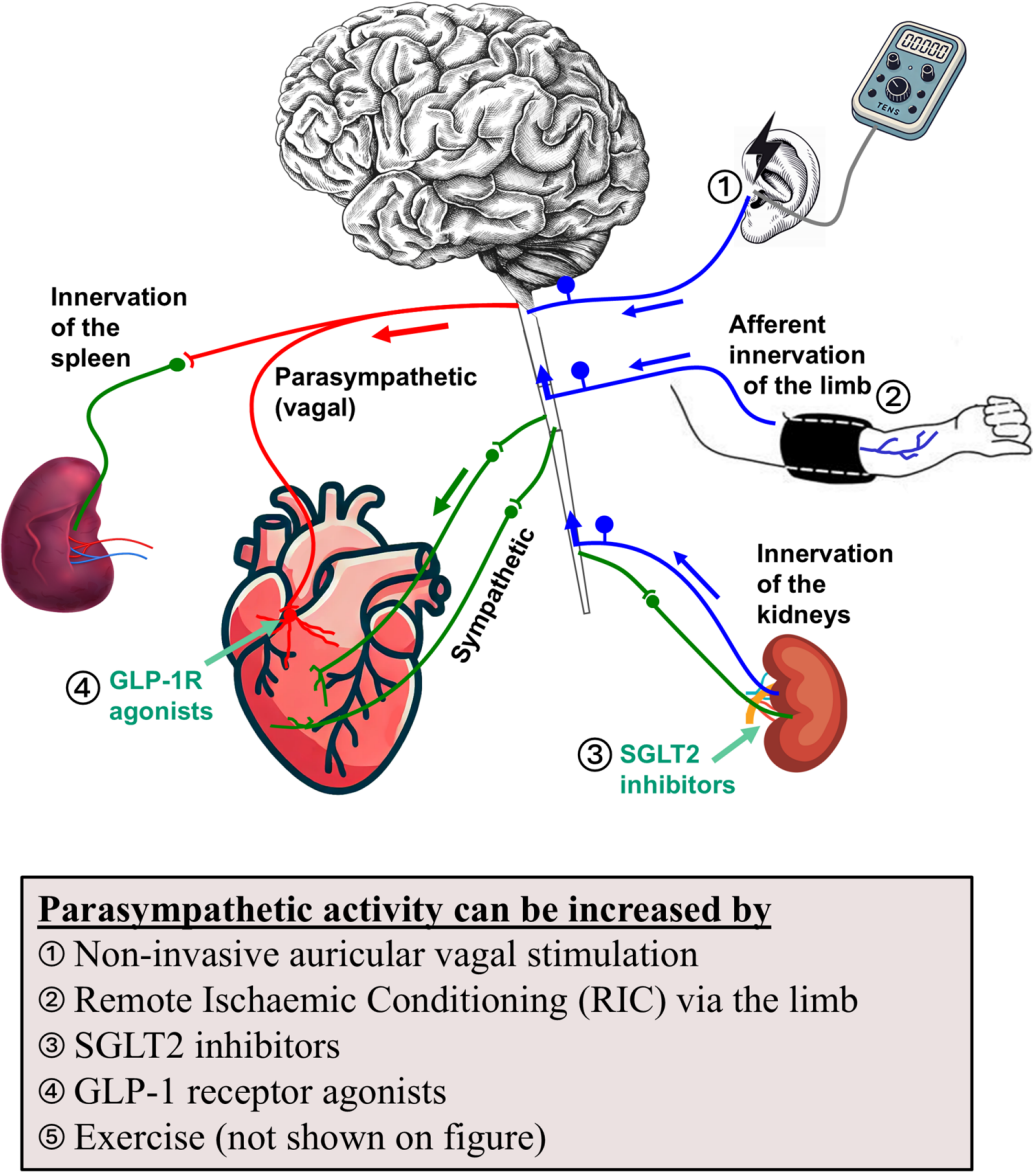
Different treatments can recruit interorgan protective afferent (blue), and efferent PNS (red) signalling pathways. Sympathetic signalling pathways are shown in green

Emerging evidence suggests that the PNS could be key to protecting organs such as the heart and brain against ischaemia/reperfusion injury [5, 7, 8, 47]. One example is RIC, which is thought to stimulate an *immediate* neural pathway (reflex), comprised of afferent signals from the limb to the PNS centres in the brain (dorsal vagal motor nucleus, DVMN), with reflex activation of efferent parasympathetic PNS signalling to the heart [85, 86], brain [5], and also to the spleen and intestinal tract [5, 68, 69]. Another example is exercise which increases vagal parasympathetic activity [35, 63] and establishes robust cardioprotection against ischaemia/reperfusion injury [87]. Interestingly, the incretin hormone GLP-1 is also involved in PNS signalling [5, 7] (Fig. 2). It has been demonstrated that combined femoral and sciatic nerve transection reduces the protective effects of RIC in rats undergoing I/R [4, 70] and vagotomy also abolishes the effects of RIC [5, 68]. Evidence to suggest that SGLT2 inhibitors may elicit some of their systemic effects by modulating renal nerve afferent activity was reported by Daniele et al. [19]. In this study, a compensatory, SGLT2 inhibitor-induced increase in hepatic glucose production was observed in patients only in conditions of intact innervation of the kidneys.

Given the aforementioned benefits of GLP-1 agonists and SGLT2i in CMK disease, it is tempting to hypothesise *that PNS involvement may underpin many currently successful cardioprotective therapies (including treatments with SGLT2 inhibitors & GLP-1 agonists), *via* afferent autonomic and somatosensory pathways and increased activity of vagal parasympathetic innervations of various organs such as the gut.*

This prompted discussion on how best to translate these findings to the clinical setting. In humans, PNS function can be determined, by the analysis of heart rate variability (HRV) or the assessment of heart rate recovery after peak exercise [35]. It was suggested that reduced vagal activity and autonomic dysfunction, highly prevalent in the patient population may offer an explanation as to why clinical trials of RIC have resulted in neutral outcomes, given the known adrenergic drive experienced by patients with ST-elevation MI [80]. Vagal parasympathetic activity decreases with age and could be severely diminished or even absent in many disease states, rendering many patients unable to recruit vagally mediated mechanisms in response to RIC [35, 55]. For example, many diabetic patients have underlying peripheral neuropathy secondary to diabetes, which is likely to directly compromise signal transmission in vagal neural pathways [36].

In respect to the protective role of PNS activation, it was noted that ultrasonic stimulation of the spleen is able to induce acute renal protection in patients with acute kidney injury [14]. This approach may be translatable to the heart, although these studies have not yet been performed.

## Novel methods of activating cellular pro-survival pathways

The major signalling pathways to organ protection by drugs and procedures (such as RIC) involve activation of signalling pathways such as the RISK (reperfusion injury salvage kinase) pathway and SAFE (survivor activating factor enhancement) pathway [45, 65, 91, 96]. The RISK pathway [91] involves the phosphorylation of AKT leading to reduced opening of the mitochondrial permeability transition pore, MPTP [91, 96].

Importantly, there are three class I isoforms of PI3K: PI3Kα, PI3Kβ and PI3Kδ. It has been demonstrated that activation of the PI3Kα isoform appears to be a pre-requisite to successful cardioprotection and the delayed opening of the MPTP [90]. Moreover, certain growth factors, such as insulin, can activate this PI3Kα-related signalling pathway and protect the heart [90]. Despite these promising results in vivo, patients with chronic diseases such as those with type-2 diabetes mellitus are often resistant to cardioprotective strategies due to receptor desensitisation [36]. These findings have also been corroborated in studies of human atrial muscle [94]. Therefore, other means of activating intracellular signalling pathways (for example, bypassing the cell surface receptors) are required.

As RIC involves PI3Kα (an integral part of the RISK pathway of cardioprotection) [96], novel PI3Kα agonists may offer a direct solution to the RIC clinical conundrum. Still under development, the emerging compounds can directly enter the cardiomyocyte, bypassing cell surface receptors. Hence, they are an attractive means of organ protection, and may be more effective than receptor-mediated approaches in patients with co-morbidities, such as type-2 diabetes mellitus and peripheral vascular disease [34].

In this regard, investigators at UCL developed a novel PI3Kα activator that can enter the cardiomyocyte, eliminating the need for receptor activation of the RISK pathway. This activator, named ‘UCL-TRO1938’, was shown to be cardioprotective in models of ischaemia/reperfusion, both in vivo and ex vivo (in Langendorff hearts), and was found to stimulate neuronal growth after neuronal crush injury as reported recently in Nature [34]. To support the general strategy of using drugs that bypass cell surface receptors to directly activate cardioprotective pathways, it was *suggested that agents such as UCL-TRO1938 should be investigated in other settings such as ischaemic stroke and in the setting of advanced age and co-morbidities such as CKD, diabetes, metabolic syndrome and hypertension*.

Another novel method was discussed related to the importance of extracellular vesicles (EVs) as agents to activate protective pathways in a range of organs [16, 20]. However, the story may be more complex. It is increasingly realised that the source of EVs is especially important in their function. For example, whilst EVs can be cardioprotective, they may also induce cardiac dysfunction when released by the brain following ischaemic stroke [15]. EVs released by platelets, in particular, have previously been shown to simultaneously protective and deleterious, in a concept also known as the ‘platelet paradox’ [20, 58]. Furthermore, co-morbidities such as diabetes can impair the signalling function of EVs [22]. Further work is required to ensure that the source from which they derive will allow for optimal cellular protection [20, 21]. In addition, characterisation of the content of EVs may similarly reveal what confers their adaptive or maladaptive effects.

## New directions in remote ischaemic conditioning (RIC)

Previous workshops have explored in detail recommendations with respect to the design of new clinical trials of cardioprotection, and in particular, further trials of RIC in the clinically ‘high-risk’ STEMI patient cohort [10, 39]. Several commentaries on the outcome of the CONDI-2/ERIC-PPCI trial [38] highlighted that fortunately the overall mortality in patients with STEMI presenting in the developed world for primary PCI is extremely low (often < 5%) [13, 39, 50, 51], *and therefore, the query has been raised: are we looking for a cardioprotective solution to a problem which does not exist (in this low-risk patient population) *[39]? In response to this key question, several further clinical trials are being undertaken to investigate the effects of RIC in high-risk STEMI patients: (1) in those presenting to multiple centres in Africa prior to thrombolysis (where PPCI is not routinely available, and STEMI 30-day mortality can be much greater, RIC AFRICA TRIAL [71], and (2) in those STEMI patients presenting to multiple centres in Germany with cardiogenic shock and/or high-risk features, (RIP-HIGH TRIAL, NCT04844931). With respect to high-risk patients, an early overview of the PERFUSION ACS registry, a preliminary study to the RIC AFRICA trial, has shown that acute coronary syndrome-associated major adverse cardiovascular events (MACE) within the region at 12 months can reach between 30 and 50% [72]. In the same study (of which 50% of patients had acute STEMI), 1-year all-cause mortality was calculated as being over 20% [72], indicating that the patients in RIC AFRICA belong to a ‘higher risk’ cohort, and may benefit the most from a very safe, non-invasive and cost-effective treatment such as RIC prior to treatment with thrombolysis, when PCI is not available.

With respect to cardiac versus all-cause mortality, there was debate as to which endpoint is preferable. Recent clinical trials have increasingly focussed on cardiovascular mortality, as opposed to all-cause mortality. This may not be ideal, as the focus on cardiovascular mortality could obscure broader safety signals related to all-cause mortality, particularly in patients with complex conditions who may be at risk of other causes of death. For example, the REWIND trial of the GLP-1 receptor agonist dulaglutide showed reductions in the composite outcome of MACE, but did not demonstrate a statistically significant reduction in all-cause mortality [32].

Although it cannot be denied that limiting infarct size is important, discussants recognised that independent prognostic value for STEMI patients can be obtained from examining the extent of microvascular obstruction and intra-myocardial haemorrhage (collectively defined as microvascular injury or MVI) [12, 23, 23, 25, 43, 56].

## The STEMI patient with MVI: a key opportunity for further research

**Fig. 3:**
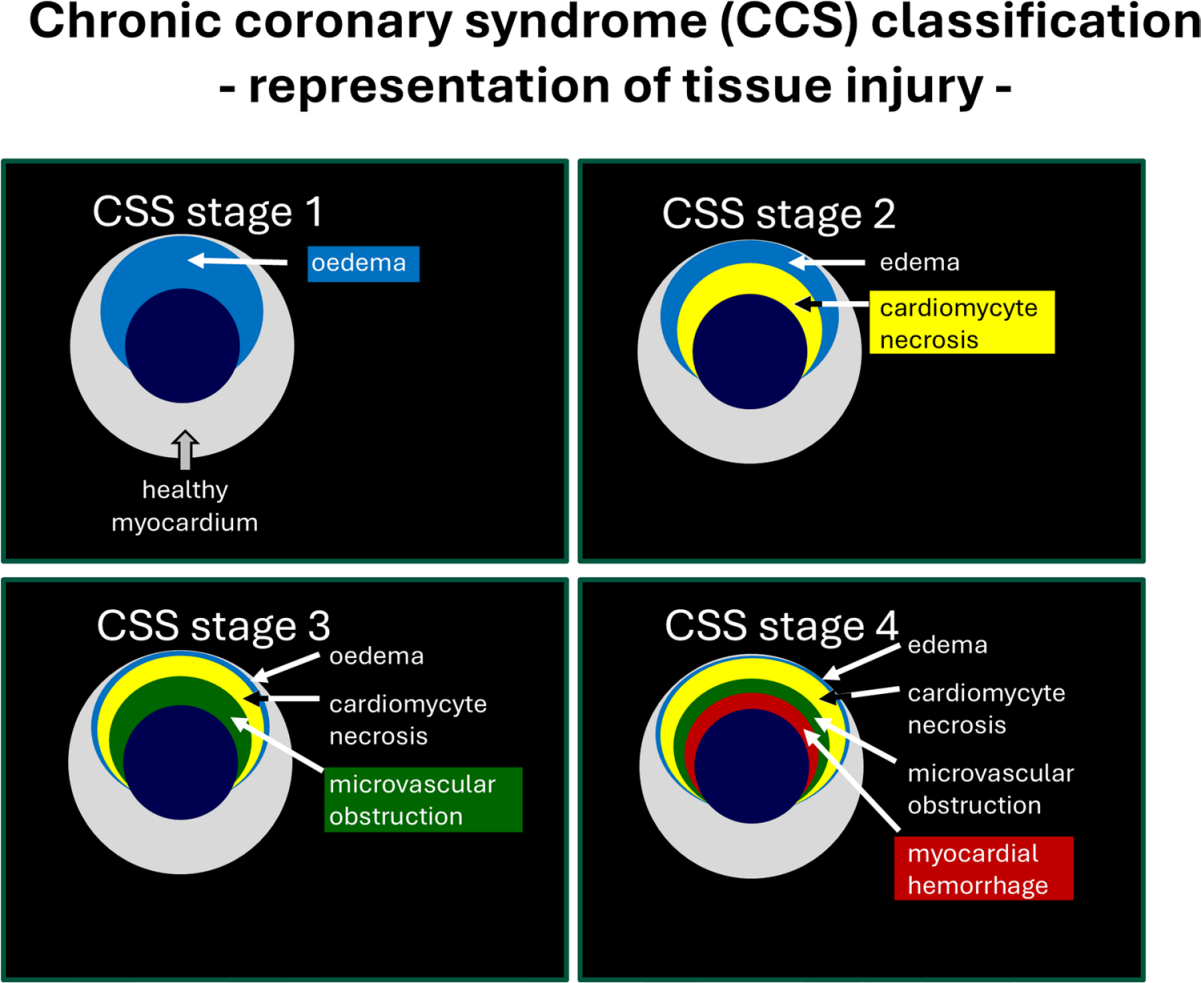
A new proposal for stages of cardiac tissue injury, including infarct, and microvascular injury. Adapted from Kumar et al. Can J Cardiol 40:1–14(2024)

Clinicians often refer to the pathological process of MVI on a continuum, with the term, ‘no re-flow’ to describe the angiographic phenomenon of persistent coronary vessel occlusion following successful opening of an obstructive athero-thrombotic coronary lesion, in the cardiac catheter laboratory [43]. MVO and MVI can also be defined using cardiac magnetic resonance imaging (MRI), and are demonstrated by regions of early gadolinium enhancement and by areas without contrast wash-in surrounded by the delayed enhanced area [53]. In pre-clinical studies, MVO is demonstrated by regions of myocardium that do not demonstrate fluorescence under UV light after reperfusion in vivo following administration of Thioflavin S dye (the latter method described by Robert Kloner in the 1970s) [61]. The clinical relevance of MVO was further supported in a pooled analyses of seven randomised clinical trials which concluded that the presence and extent of MVO (measured by MRI) after primary PCI in STEMI patients was strongly associated with mortality and hospitalisation for heart failure [23]. Despite early interest in no reflow and MVO in the pre-clinical field [61], no gold standard therapies exist to treat this important clinical problem. This has been highlighted previously by an important meta-analysis by Heusch et al. in 2019 [43] which did not identify any clear benefits for MVO outcomes from thrombolysis, mechanical intervention, adenosine or nitrate therapies [43]. Moreover, adenosine as an intervention for no reflow was associated with harmful outcomes in the prior REFLO-STEMI trial [78]. Possible exceptions to this include metoprolol as an intervention with respect to myocardial salvage index (as a result of the previous METOCARD-CNIC TRIAL) [31, 54] (and Nicorandil with respect to combined TIMI flow and MRI outcomes in the more recent CHANGE trial [82, 88]. The Canadian Cardiovascular Society has recently re-classified the severity of myocardial infarction with respect to cardiomyocyte and coronary circulation injury to reflect cardiomyocyte cell death, MVO and intra-myocardial haemorrhage in combination (grade IV) as representing the most severe form of injury [64] (Fig. 3). It was discussed how best to define a successful drug for MVO, both in pre-clinical and clinical research. *For example, should one consider an improvement of MVO parameters as being sufficient, or should one also consider changes in infarct size, intra-myocardial haemorrhage, and other catheter laboratory indices* [43]?

The participants acknowledged that the pathology of MVI involves a complex interplay of micro-emboli [60], immune-platelet complexes, vascular stasis, peripheral oedema causing external compression, vascular smooth muscle cells (VSMC) spasm and pericyte constriction [18], and an increase in capillary membrane permeability, leading to capillary rupture [37, 42, 42, 62]. In addition, it was highlighted that MVO is highly dynamic and dramatically changes within days after infarction complicating the interpretation of observational data and of the studies using interventions and different timing [26]. A main discussion point was the need for new therapeutic strategies in the field and the evidence for the contribution of pericyte involvement in MVO following myocardial ischaemia/reperfusion [18]. Pericytes are known to constrict the capillary bed both during ischaemia and during reperfusion, which can lead to irreversible vascular change and obstruction of blood flow [57]. *The group considered that focus should be not only on protecting the endothelium, but attention should be given to mural cells such as the vascular smooth muscle cells (VSMC) and pericytes*.

A new proposal was the potential for a role of Rho Kinase (ROCK) in STEMI-associated MVI, based upon recent pilot data from pre-clinical studies in vivo, using a Thioflavin S model of no reflow [83]. The non-selective ROCK1/2 inhibitor, Fasudil, an effective arterial vasodilator (acting on the calcium sensitisation pathway of VSMC contraction), has been used previously both in VSMC spasm associated with cerebral haemorrhage [93] and stable angina [75]. It is yet to be re-purposed as a drug for no reflow in large-scale clinical trials. Moreover, ROCK2-specific inhibitors, such as the anti-inflammatory drug, KD025 (also used in haematological graft vs host disease), were found to reduce both MVO and myocardial haemorrhage in vivo [83].

Importantly, it was viewed that MVO does not just underlie STEMI but is an important process in cardiotoxicity (in the field of cardio-oncology). In fact, it has been shown that anthracyclines induce an early and irreversible damage to the coronary microcirculation [29].

## New considerations in cardio-oncology

The field of cardio-oncology is challenging in that it encompasses a wide range of pathological processes including cardiotoxicity-induced heart failure, premature coronary artery disease, microvascular coronary injury and spasm, cardiac inflammation, arrhythmia and thromboembolism [66, 76, 95]. Modern advances in cancer treatment have resulted in patients living longer and becoming tumour free. However, many patients subsequently suffer from cardiac side effects associated with cancer therapy [76]. The agents most commonly associated with cardiotoxicity are the anthracyclines (which cause heart failure) and radiotherapy (which causes premature coronary artery disease). The proportion of patients affected by anthracycline toxicity is estimated to be between 5 and 23% [66], and this can range from anywhere between subclinical left ventricular impairment (causing a reduction in global longitudinal strain (GLS) on echocardiography) to severe, symptomatic heart failure requiring inotropic support and mechanical circulatory support [76].

The participants discussed the need for cardioprotective strategies that would benefit this cohort of patients. Importantly, it is possible to predict which patients are at higher risk for complications of cardiotoxicity. These include those with pre-existing CVD (elevated risk) and high cumulative dose of chemotherapeutic agent (high risk). Elevated biomarkers such as troponin, cardiac myosin-binding protein C (CMyC), and B-type natriuretic peptide (BNP) might indicate that a patient belongs to the medium risk cohort [89], as demonstrated in the ERIC-ONC study [73]. This increased risk of cardiotoxicity might be further estimated with tools such as the recently published HFA-ICOS score [89]. In clinical trials of cardioprotection from acute anthracycline cardiotoxicity, the ERIC-ONC study did not describe any overall benefit from patients undergoing chemotherapy who received RIC as an intervention prior to anthracyclines vs sham group [41, 73]. Nevertheless, there was a significant increase in both troponin and CMyC, indicating possible cardiac injury [73].

The upcoming RESILIENCE trial, which is currently ongoing across 6 countries including Spain, Portugal, Germany, Denmark, Netherlands, and France [77], was also discussed. RESILIENCE is a prospective, randomised, sham-controlled clinical trial, investigating the effects of RIC prior to anthracycline chemotherapy for patients with Hodgkin’s lymphoma [77]. The primary endpoint of the study is the change in the % of left ventricular ejection fraction (LVEF%), with secondary endpoints including MACE, tumour progression, T2 mapping on cardiac MRI as an early marker of cardiotoxicity and ultrafast MRI (1 min) [33] to allow scanning of vulnerable population [28, 74]. The investigators have previously performed a key, large animal, pre-clinical study, investigating the effects of RIC as an intervention, in pigs receiving anthracyclines [30]. Here, pre-treatment with RIC significantly ameliorated reduction in % of LVEF, and attenuated left ventricular interstitial fibrosis and mitochondrial fragmentation, which was otherwise observed heavily in the sham group [30]. The RIC protocol used in the larger animal study involved 5 min of ischaemia followed by 5 min of reperfusion (3 cycles). In the RESILIENCE trial, RIC will be performed weekly throughout the entire duration of the chemotherapy period (approx. 4 months) [77]. The participants made the observation that *there have been no dedicated dose–response studies, for RIC as an intervention in this setting, and that this would be worthwhile, in order to establish a therapeutic protocol for any future planned animal and human studies.*

The workshop participants discussed the importance of developing appropriate pre-clinical models to investigate cardiotoxicity, specifically, the use of tumour-bearing models, and those with appropriate chemotherapy regimens. There was discussion of the need to distinguish the cardiotoxic effects of chemotherapy drugs from those of the tumour itself [1, 17], and this remains a currently unmet need in pre-clinical research.

One common phenomenon observed in both pre-clinical tumour-bearing animal models and humans with cancer is the concept of ‘cardiac wasting.’ This is felt to be attributable to left ventricular fibrosis and contractile dysfunction, with contributions from inflammation (TNF-α, IL-6 mediated) and oxidative stress [2]. Patient autopsies have revealed a significant reduction in cardiac mass as a result of wasting, irrespective of BMI, and these patients have greater elevations in troponin and brain natriuretic peptide (BNP) compared to cancer counterparts without cardiac wasting [2]. It is proposed that pre-clinical research should seek to gain better understanding of this process, in order to target future therapies for left ventricular impairment in cancer and cardiotoxicity. This session defined key goals for further pre-clinical research in cardio-oncology as being*: 1) the need to develop a novel cardioprotective strategy that is both advantageous against both cardiac wasting in cancer and chemotherapy-related toxicity; and 2) any cardioprotective agent should not impair tumour response to chemotherapy* [2, 41, 76].

## Conclusion

The 12th Hatter Cardiovascular Workshop identified important points and key questions for future research in the fields of cardio- and neuro-protection and the importance of systems approach to our understanding of interorgan communication. These are summarised in the following 10 points:

1. Animal models that reflect the clinical scenarios in patients with CKD should be developed to improve translation. Models should include CMK and investigate the role of co-morbidities such as the metabolic syndrome, diabetes and CKD in cardiac protection.
2. Recent pharmacological developments (e.g., SGLT2i, GLP1ra) suggest that an integrative, multi-organ approach is achievable in developing treatments for complex syndromes with multiple co-morbidities. As such, it is important to not only use the appropriate experimental models, but also to examine the effects of therapies and drugs on interorgan communication in those models.
3. Individuals exhibit different likelihoods of developing diseases even if they have the same risk factors, suggesting there may be patients who are susceptible and who are not susceptible to a particular disease. If so, how can they be identified? Similarly, are there “responders” and “non-responders” to *therapies,* who can be identified in order to optimise and personalise treatment [48]?
4. As highlighted in the report, clinical trials should include endpoints of both cardiovascular mortality and all-cause mortality.
5. New concepts were discussed that highlighted the potentially critical role for the parasympathetic nervous system in mediating cardio- and neuro-protection induced by RIC, exercise, and novel anti-diabetic drugs such as SGLT2 inhibitors and agonists of GLP-1 receptors.
6. The concept of a non-receptor-based means of activating intracellular signalling pathways (thereby bypassing the cell surface receptors) was discussed, in which agents could be investigated in advanced age and co-morbid settings such as CKD, metabolic syndrome, diabetes, and hypertension where cell surface receptors may be compromised.
7. Extracellular vesicles show potential for cellular protection in the heart and the brain, but significant research is required to understand how co-morbidities impact their effectiveness.
8. RIC has been shown to be an attractive non-invasive means of organ protection; however, in large outcome trials, it has not yet been shown to be effective. Modern medicine has shown that patients in the setting of STEMI have limited injury following primary PCI; however, RIC may still be effective in high-risk populations.
9. An additional important clinical target which is often neglected in the I/R setting is that of coronary microvascular obstruction and injury. As yet, there is no specific therapy to treat such patients who develop microvascular injury, and new concepts such as targeting pathways involving rho kinase were discussed.
10. In the setting of cancer and chemotherapy-related cardiac damage, there is a need to develop novel cardioprotective strategies that are advantageous against cardiac injury but do not impair tumour response to chemotherapy.
